# The Glia Response after Peripheral Nerve Injury: A Comparison between Schwann Cells and Olfactory Ensheathing Cells and Their Uses for Neural Regenerative Therapies

**DOI:** 10.3390/ijms18020287

**Published:** 2017-01-29

**Authors:** Matthew J. Barton, James St John, Mary Clarke, Alison Wright, Jenny Ekberg

**Affiliations:** 1 Menzies Health Institute Queensland, Griffith University, Nathan QLD 4111, Australia; j.stjohn@griffith.edu.au; 2 Clem Jones Centre for Neurobiology & Stem Cell Research, Griffith University, Nathan QLD 4111, Australia; jekberg@bond.edu.au; 3 Griffith Institute for Drug Discovery, Griffith University, Nathan QLD 4111, Australia; m.clarke1@griffith.edu.au; 4 Faculty of Health and Medical Science, Bond University, Robina QLD 4226, Australia; alison.wright@griffith.edu.au

**Keywords:** nerve-injury, nerve-regeneration, Schwann-cell, olfactory-ensheathing-cell, glia

## Abstract

The peripheral nervous system (PNS) exhibits a much larger capacity for regeneration than the central nervous system (CNS). One reason for this difference is the difference in glial cell types between the two systems. PNS glia respond rapidly to nerve injury by clearing debris from the injury site, supplying essential growth factors and providing structural support; all of which enhances neuronal regeneration. Thus, transplantation of glial cells from the PNS is a very promising therapy for injuries to both the PNS and the CNS. There are two key types of PNS glia: olfactory ensheathing cells (OECs), which populate the olfactory nerve, and Schwann cells (SCs), which are present in the rest of the PNS. These two glial types share many similar morphological and functional characteristics but also exhibit key differences. The olfactory nerve is constantly turning over throughout life, which means OECs are continuously stimulating neural regeneration, whilst SCs only promote regeneration after direct injury to the PNS. This review presents a comparison between these two PNS systems in respect to normal physiology, developmental anatomy, glial functions and their responses to injury. A thorough understanding of the mechanisms and differences between the two systems is crucial for the development of future therapies using transplantation of peripheral glia to treat neural injuries and/or disease.

## 1. Introduction

The central nervous system (CNS; brain and spinal cord) has a very limited capacity for functional regeneration after injury. In contrast, the peripheral nervous system (PNS; all other nervous tissue) can regenerate; the extent to which depends on the size and nature of the injury. One subsection of the PNS that is particularly adept at regeneration is the olfactory nerve that continuously regenerates throughout life. Within the PNS there are two types of glial cells that play pivotal roles in restoring function after nerve injury, these are Schwann cells (SCs) and olfactory ensheathing cells (OECs). The majority of nerves in the PNS (hereafter referred to as the general PNS) are populated by SCs, whereas the peripheral nerve that transmits the special sense of smell; the olfactory nerve (hereafter referred to the olfactory nervous system), contains OECs. These two glial cell types share many morphological, embryological and functional similarities; however, they also exhibit distinct functional differences relating to their respective neural regenerative abilities after injury. This review will explore these similarities and differences, and highlight key unique features that may be utilised for future neuronal regeneration therapies.

## 2. Anatomy and Histology

### 2.1. The Olfactory Nervous System

**Figure 1:**
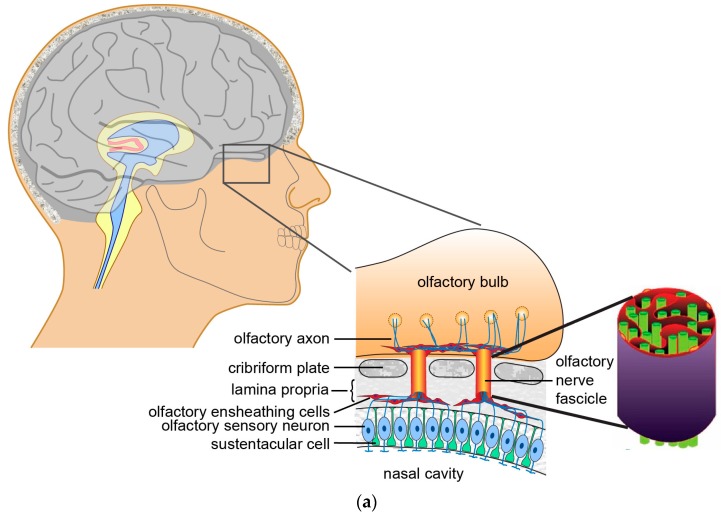

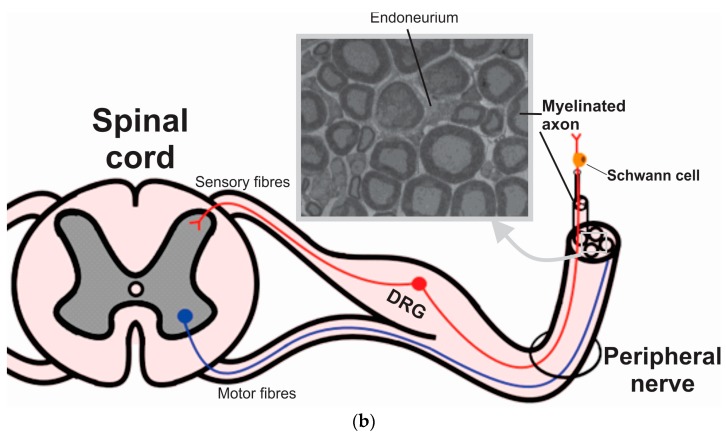
Anatomy of olfactory system and the general peripheral nervous system (PNS): (**a**) Olfactory nervous system: Schematic image of a sagittal section of the olfactory system, where the cell bodies of olfactory neurons (blue) are localised in the nasal mucosa, their dendrites project into the nasal cavity and their axons are fasciculated by olfactory ensheathing cells (OECs) (red) from the nasal mucosa to the olfactory bulb; (**b**) General PNS: Transverse section of a mixed nerve projecting out of the spinal cord. SCs surround individual axons (sensory axons shown in red, motor axons shown in blue), which form small bundles (fascicles) that together constitute the nerve. Cell bodies of sensory neurons are located in the dorsal root ganglion (DRG), whereas the motor neuron somas are found in the ventral horn within the grey matter of the spinal cord.

The primary olfactory nervous system consists of two olfactory nerves (cranial nerve 1) in the PNS, and their respective olfactory bulbs in the CNS. The olfactory nerve extends from the olfactory epithelium in the nasal cavity to the olfactory bulb in the brain (Figure 1a). The neurons that comprise the nerve are olfactory sensory neurons, which detect different odorants depending on their olfactory receptor profile. Humans have up to 400 different functional olfactory receptors that detect a multitude of distinct odorants molecules [1]. The neuronal cell bodies are localised in the olfactory epithelium, which occupies 1–2 cm of the roof of the nasal cavity [2], and their dendrites with the olfactory receptors extend into the nasal epithelium where odorant detection takes place (Figure 1a). Olfactory axons project from the cell bodies through the underlying lamina propria and, form small fascicles (bundles) that project through the cribriform plate of the ethmoid bone, all the way to the olfactory bulb (Figure 1a). Together, these fascicles constitute the highly branched peripheral olfactory nerve. OECs do not myelinate olfactory axons; instead, each OEC encloses several olfactory axons which together constitute one fascicle (Figure 1a) [3]. Here, OECs form a stable channel which forms a strong mechanical supportive conduit for the axons [4,5]. OECs express a number of ECM proteins (laminin, fibronectin, neural/glia antigen 2 (NG2), galectin-1 etc.) which have been suggested to play pivotal roles in neurogenesis, neuronal survival and regeneration [6,7].

When the axons reach the olfactory bulb, they de-fasciculate and re-organise in the outer nerve fibre layer of the bulb, and then when reaching the inner nerve fibre layer, re-fasciculate axons from neurons expressing the same olfactory receptor subtype. The pure fascicles terminate in specific glomeruli deeper within the bulb, dedicated to a certain olfactory receptor profile. Within the glomeruli, the neurons then communicate with higher order olfactory neurons ultimately leading to the olfactory cerebral cortex [8].

### 2.2. The General PNS

Peripheral nerves are elaborate organs, composed of nervous tissue, connective stroma and a complex vascular network [9]. Most peripheral nerves contain both sensory (afferent) and motor (efferent) axons with variable axon diameter from 1 µm (unmyelinated pain-sensing neurons) to 20 μm (large, myelinated motor axons) [10]. The cell bodies of peripheral motor neurons and sensory neurons are localised in the anterior horn of the spinal cord and dorsal root ganglia (DRG), respectively (Figure 1b). Peripheral axons and their associated SCs are surrounded and supported by a stratification of connective tissue architecture [11]. The innermost layer surrounding each individual axon is the endoneurium (Figure 1b), which contains endoneurial extracellular matrix (ECM) consisting predominately of collagen bundles, providing an important protective channel for the axon. Although endoneurial fibroblasts secrete ECM proteins into the endoneurial tube [12], SCs are the chief orchestrator in synthetizing collagen for both the endoneurium and perineurium [13]. The ECM contains insoluble components (collagen, fibronectin, and laminin) that provide mechanical support for the axon and physically prevents conduction interference between adjacent axons [13]. Groups of endoneurial tubes are formed into larger fascicles named the perineurium; a mechanically strong sheath enclosed by a basal lamina and interspersed by longitudinal collagen fibres [9]. The epineurium is the outermost loose connective tissue layer, consisting of a more sparse arrangement of collagen fibres and adipose tissue, which encapsulate the perineurial bundles and nutritive blood vessels to form a single nerve.

## 3. Embryology

### 3.1. The Olfactory System

The olfactory sensory neurons of the peripheral olfactory system arise from the olfactory placode and extend axons to merge with the evaginating telencephalon to form the olfactory bulb. During the embryological process of neurulation, a population of cells known as neural crest cells arise [14]; which appear consistent throughout all vertebrates [15]. Although OECs migrate out from the olfactory placode region, their origin is from these neural crest cells which migrate to the olfactory mucosa to establish the OEC precursor population [16]. OECs first appear in the olfactory tract (embryonic day 10.5 [17] in mice) and they migrate with the extending axons to merge with the developing olfactory bulb where the OECs are restricted to the nerve fibre layer of the olfactory bulb; thus, OECs are in close contact with olfactory axons all the way from the basal olfactory epithelium of the nasal cavity to the outer layer of the olfactory bulb [5]. During development, OECs ensheathe multiple unmyelinated olfactory sensory axons within the same nerve fascicle en route to the olfactory bulb, then when the axons enter the nerve fibre layer the OECs aid the defasciculation, sorting out and targeting of the axons to their appropriate topographic locations in the deeper layer of the olfactory bulb [18].

### 3.2. The General PNS

Neural crest cells differentiate into many cells that are not part of the PNS (melanocytes, craniofacial mesenchyme, cardiac-septal smooth myocytes, etc.) and into PNS cells such as sensory neurons, satellite cells and SCs [14]. Thus, OECs and SCs both originate from the neural crest. Before SCs can populate peripheral nerves, they must proceed through three stages of development; (1) SC precursors (SCPs); (2) immature SCs (iSCs); and (3) myelinating (mSCs) or non-myelinating (nmSCs) SCs [19]. Firstly, neural crest cells will develop into SCPs (14–15 of gestation in rats; days 12–13 in mice; and around the 12th week in humans [20]). Shortly thereafter, these SCPs will differentiate into iSCs where they then migrate into nerve fascicles and organise themselves as single cells or cords to assist with axonal sorting [20]. SCPs are most important in providing trophic support for developing neurons and preventing abnormal synaptic connections between the developing neurons and their targets [14]. The iSCs will continue to penetrate the axon bundles and separate axons with their cytoplasmic extensions, through a process called radial sorting [19]. iSCs that are in contact with small-diameter axons (<1 µm), will differentiate into mature nmSCs, which enwrap several unmyelinated axons into a bundle termed a Remak bundle [19]. In contrast, iSCs that establish a 1:1 ratio with larger (>1 μm) diameter axons will become mature mSCs. When encountering a medium-to-large sized axon, the lamellipodial membrane protrusions from the mSC interact with the axon, initiating the myelinating process. As the thickness of the myelin envelope increases, so does the conduction speed of the nerve fibre, due to the insulating properties of the fatty membranous myelin layer.

## 4. Homeostatic Roles

### 4.1. The Olfactory Nervous System

The olfactory nerve is unique in that it continuously renews itself. Olfactory neurons are constantly exposed to the external environment due to their anatomy, and remain viable for approximately one month (in rodents), where 1%–3% of neurons are turned over daily [21]. Thus, the olfactory nervous system is constantly undergoing neurogenesis, and this unique feature is now largely attributed to the presence of OECs. OECs exhibit unique growth-promoting and migratory properties, and other biological functions directly related to neuronal survival and axonal extension. The olfactory nerve is almost completely devoid of macrophages, and macrophages are not in direct contact with olfactory axons. Instead, OECs are the primary immune cells of the olfactory nerve, responsible for clearing both the axonal debris resulting from the olfactory nerve turnover and invading microorganisms [4]. Olfactory neuron dendrites extend directly into the nasal cavity and are constantly exposed to microorganisms, and thus, OECs are considered crucial for protecting the CNS from microbial invasion via the olfactory nerve [4,22,23]. OECs are not one uniform population of cells; there are at least five subpopulations with distinct functions and anatomical locations [24]. OECs in the olfactory nerve promote axonal fasciculation whilst OECs from the nerve fibre layer of the olfactory bulb mediate defasciculation, sorting and refasciculation of axons, and directing the axons to their appropriate glomerular target [3,24]. OECs exhibit highly motile lamellipodial protrusions that are crucial for OEC migration, cell-cell contacts and phagocytic activity. The difference in function between OEC subpopulations is directly related to the activity and behaviour of lamellipodial waves; when these waves are inhibited pharmacologically, the heterogeneity disappears [24]. In summary, OECs exhibit important structural, growth-promoting and immunological roles essential for the homeostasis of the olfactory nervous system.

### 4.2. The General PNS

The architecture of the general PNS is a stable structure mediated by SCs. mSCs associate closely with motor neurons and larger diameter sensory neurons, conversely nmSCs typically associate with small sensory neurons (i.e., C fibers); nevertheless both mSCs and nmSCs provide support in the form of contact, myelin and neurotrophins [25]. Whilst nmSCs do not myelinate using a myelin-sheath, C-fibers are sub-divided into bundles via membrane extensions from nmSCs [25,26]. As discussed earlier, axons with large diameters prompt myelination during their development. Conversely however, myelination can actually increase the diameter of axons. Due to the tightly wrapped myelin layer surrounding individual axons, mSCs are in closer physical proximity with their axons than nmSCs. Neurons with a close axo-glial relationship have larger axonal diameters, due to the release of myelin-associated glycoprotein (MAG) from mSCs, which supports neuronal connection stability and increases axonal diameter [27]. MAG also attenuates the reparative responses to injury by inhibiting the formation of growth cones and axonal outgrowth [27,28]. Myelin-lipid homeostasis is also an integral component of PNS stability. Metabolically, SCs play an important role in this process through acetyl-COA carboxylase phosphorylation, which increases lipid production in myelin and decreases lipid oxidation. Maintaining a high lipid concentration in the myelin layer ensures normal saltatory conduction, and neuron integrity [29,30]. The effects of SCs are strongly directed towards assisting the function and maintaining the stability of the neuronal connections and architecture within the PNS. The close relationship between SCs and their neurons promote survival and stability particularly through protein binding and myelin metabolism.

## 5. Response to Injury

Peripheral nerves are commonly damaged after traumatic injuries, infections and surgery. In the general PNS, the capacity for nerve regeneration is dependent on the extent of injury, where large nerve injuries can lead to permanent disability. In the United States, ~20 million people are currently living with permanent nerve damage [31], and over 360,000 people per annum sustain peripheral nerve damage just in their upper extremities [32]. Olfactory nerve injuries regenerate completely over 1–2 months [33], unless the olfactory bulb or other CNS areas involved in olfaction are damaged, resulting in anosmia or dysosmia [34]. Many of the injuries to nerves in the general PNS result in axonal damage whilst the cell bodies, which are localised in the DRG (sensory neurons) or spinal cord/brain (motor neurons), are preserved [35]. In contrast, olfactory nerve injury often causes both axonal damage and death of the entire neuron, including the cell body [8,36,37]. Thus, neurons in the PNS must regenerate their axons and re-innervate their targets whilst olfactory neurons must be replaced with new neurons originating from progenitor cells, which then extend axons towards the CNS and re-innervate the olfactory bulb. In both the general PNS and the olfactory nerve, the first stage of regeneration involves extensive clearance of axonal debris followed by regeneration and re-innervation. Glial cells and other cells such as macrophages and fibroblasts play crucial roles in these processes [3]. The remaining sections of this review will explore the glia response in both the general PNS and the olfactory nervous system following injury; specifically, their respective role in clearance, immune system modulation and growth-support signalling that promotes nerve regeneration.

### 5.1. The Olfactory Nervous System

Small injuries to the olfactory nerve are common, but even large-scale olfactory injuries can still lead to complete restoration of the sense of smell, unless the olfactory bulb or other CNS structures involved in the sense of smell are damaged [8]. Damage to the olfactory nerve or bulb can result in dysfunction or loss of the sense of smell, depending on the severity of the injury. Head trauma and upper respiratory tract infections are common causes of dysfunction [38,39], as well as trans-sphenoidal surgery to remove tumours in the basal region of the brain, such as pituitary tumours [40]. Although there are few physical disabilities associated with impaired olfaction the psychological impacts can be particularly detrimental to one’s quality of life. From 1000 patients surveyed about their olfactory dysfunction there were numerous reports of social isolation and anhedonia, the loss of enjoyment from previously pleasurable activities [41].

#### 5.1.1. OEC Response to Injury

After serious injuries to the olfactory tract, such as olfactory bulb ablation, OECs proliferate and migrate ahead of regenerating axons [42]. After experimental peripheral injury to the olfactory epithelium with zinc sulphate or methimazole, OECs also migrate towards the bulb, undergo morphological changes and increase their level of phagocytosis [4,22,43]. One study, however, showed that OECs did not proliferate or migrate after olfactory nerve axotomy, but instead maintained open channels through which new axons could extend [44], suggesting that the behaviour of OECs is adaptable to the type of nerve injury.

#### 5.1.2. OEC Clearance of Debris

Axonal debris is continuously being generated in the intact olfactory nerve due to the constant regeneration of olfactory sensory neurons. Macrophages are largely absent from this nerve, and are not in direct contact with axon fascicles. Instead, it is OECs that continuously clear the debris [22]. As discussed earlier, OECs have been identified as the primary innate immunocyte of the olfactory nervous system [45]. Even after olfactory nerve injury, OECs are the main cells responsible for debris clearance, and macrophage recruitment to the area is very limited [22].

OECs also readily phagocytose and degrade bacteria [23], a very important immune function since the olfactory nerve constitutes a direct path from the nasal cavity into the brain. Toll-like receptor 4 (TLR4), which is expressed by OECs, is crucial for phagocytosis of *Escherichia coli* [46]. The molecular mechanisms behind OEC-mediated phagocytosis of axonal debris, however, are to date largely unknown. In vitro experiments of OEC phagocytosis have revealed that the phagocytic activity of OECs can be stimulated. One such activator of phagocytic activity is the alkaloid curcumin, a component of turmeric with neuroprotective properties, which at low concentrations stimulates OEC-mediated phagocytosis of axonal debris by 10-fold [47] likely by involving mitogen-activated protein (MAP) kinases [47]. The importance of OEC phagocytosis is highlighted by the comparison with SCs where curcumin does not stimulate phagocytosis of axonal debris by SCs. This suggests that there are fundamental differences in the cellular and molecular mechanisms underlying responses to cellular debris between the two cell types [48]. These differences may be crucial for the difference in regenerative capacity between the primary olfactory nervous system and the general PNS.

#### 5.1.3. OEC’s Regulation of Inflammation/Immune Response

**Figure 2:**
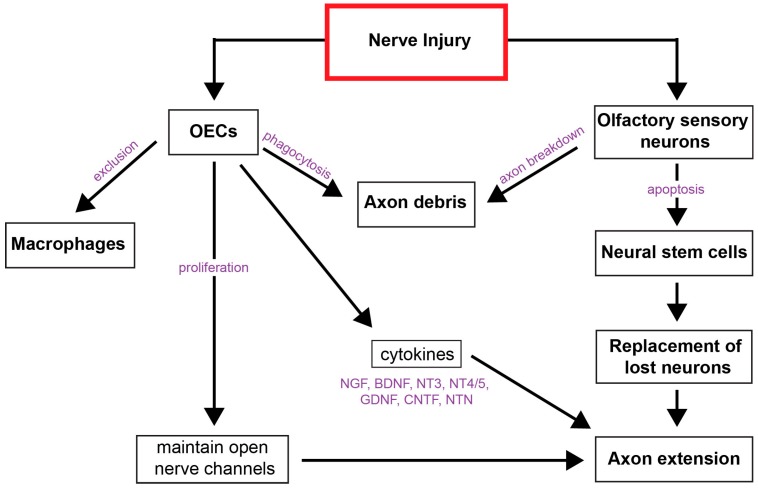
Overview of olfactory ensheathing cell response to olfactory nerve injury. (Arrows connect sequential events, NGF, nerve growth factors; BDNF; brain derived neurotrophic factor; NT, neurotrophin; GDNF, Glial cell-derived neurotrophic factor; CNTF, Ciliary neurotrophic factor; NTN, neurturin).

OECs in the primary olfactory nervous system do not produce cytokines that attract macrophages after injury (Figure 2). Leukemia inhibitory factor (LIF) and Tumour necrosis factor (TNFα) have been detected in the olfactory system; however, these cytokines are produced by cells other than OECs, and their expression does not increase after injury [49,50]. LIF is produced by the olfactory sensory neurons [51] and has been linked to neuron development and maturation. In LIF knockout mice, a greater population of mature olfactory sensory neurons are observed [52]. LIF also promotes neural progenitor proliferation after injury in the olfactory epithelium of mice [51], by inducing nitric oxide synthase [53]. TNFα is secreted by olfactory sustentacular cells, the non-glial supporting cells of the lamina propria that surround olfactory receptor neurons and provide the external barrier to the epithelium. Here, TNFα production can be induced in inducible olfactory inflammation (IOI) mice. These transgenic mice, used to model olfactory inflammation, showed that TNFα expression causes olfactory receptor neuron death after 28 days but the damage is reversible once TNFα expression ceases, and complete regeneration ensues [54]. In this animal model, a large number of macrophages infiltrated the olfactory submucosa during TNFα expression, which resulted in selective death of olfactory sensory neurons. Demonstrating that factors produced by macrophages are harmful to olfactory neurons [54], further strengthening the notion that OECs are the primary immune cells in the healthy and injured olfactory nervous system.

#### 5.1.4. OEC’s Growth-Support Signaling

OECs are responsible for creating an environment conducive to neuron growth and axon regeneration by producing neurotrophins. Neurotrophic factors promote neuron growth and survival. OEC populations express mRNA for nerve growth factor (NGF), brain-derived neurotrophic factor (BDNF), neurotrophin 3 (NT-3), neurotrophin 4/5 (NT-4/5), neuregulin (NRG) ciliary neurotrophic factor (CNTF), neurturin (NTN), and glial-derived growth factor (GDNF) with variations of expression attributable to stress and injury [55,56]. The secretions of these factors have the potential to directly and indirectly support neuron growth through autocrine action, creating a more supportive phenotype and paracrine action directly affecting neuron growth. The main neurotrophin family NGF, BDNF, and NT-3 act on tyrosine kinase receptors (TrkA, TrkB and TrkC) respectively with a degree of affinity crossover between receptors and low affinity with p75NTR. Moreover, OECs express p75NTR, TrkB and TrkC which when bound to BDNF and NT-3 become cytoprotective and counteract neural pathology associate with transplantation [57]. OECs also express the receptors for GDNF binding—GFRα-1 and GFRα-2 [56]. Paracrine activation of other cells such as astrocytes and neurons via these neurotrophic factors can inhibit astrocytic boundary formation and stimulate neurite outgrowth in neurons [58,59]. The high levels of neurotrophins secreted from OECs exceed SCs with the exception of an injury state, such as the increase in BDNF seen following nerve transection [56,60]. The extensive range and consistent high levels of growth factor expression contribute to the environment of primary olfactory nervous system, which is conducive to continual nerve regeneration.

### 5.2. The General PNS

Nerves that are aged, sensory, or have sustained injuries closer to their soma (spinal cord and/or DRG) are less likely to survive injury and regenerate. After sustaining a crush type injury, functional recovery is probable, as the connective tissue architecture remains intact and not all axons are severed. After complete nerve transection (axotomy), however, functional recovery is harder to attain as the nerve stumps retract, creating a physical gap that regenerating axons need to navigate across to innervate their original target. Thus, if the gap is large (>20 mm) and close to the soma, chronic axotomy is expected [61,62], consequently leading to chronic SC denervation. Both chronic axotomy and SC denervation are exacerbated by the slow axon regeneration rate of ~1 mm/day [63] and axon misdirection; where axons reconnect to the wrong target.

#### 5.2.1. SC Response to Injury

In an effort to create an environment that supports and facilitates axonal growth and target reinnvervation after injury, a number of cell types will alter their gene expression; producing different phenotypes. For example, neurons with damaged axons are known to switch from a “conducting” phenotype to a “growing” phenotype [64]. Whilst, mSCs de-differentiate into nmSCs by down regulating myelinating genes and up regulating regeneration-associated genes (neurotrophins and adhesion molecules) [63]. This nmSC phenotype switch is short-lived however, where these regeneration-associated genes will be downregulated or switched off ~6 months after injury [64]. Once the nmSCs have re-differentiated into mSCs they myelinate any regenerated viable axons [65], while those SCs who do not associate with a viable axon—and receive NRG1—will ultimately die [66]. As well as switching phenotypes, SCs proliferate in response to injury [67]. This provides growth factor expression, modulates local immune system events, and perform debris clearance in processes, such as Wallerian degeneration that is all crucial for axonal regeneration.

#### 5.2.2. SC Clearance of Debris

**Figure 3:**
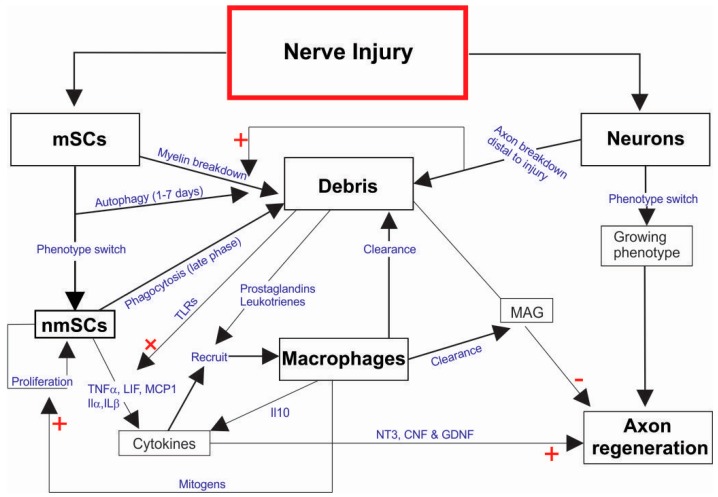
Overview of Schwann cell response to peripheral nerve injury. (Arrows connect sequential events; + denotes a positive response; − denotes a negative or inhibitory response; mSCs, myelinating Schwann cells; nmSCs, non-myelinating Schwann cells; MAG, myelin associated protein; TLRs, toll-like receptors; CNF, cytotoxic necrotizing factor; TNF, tumor necrosis factor; LIF, leukemia inhibitory factor; MCP1, monocyte chemoattractant protein-1; IL, interleukins).

Before peripheral nerve regeneration can occur, axonal and myelin debris, and other cell debris must first be cleared away. This process is a crucial part of the immediate response to peripheral nerve injury; termed Wallerian degeneration. Wallerian degeneration constitutes the axonal breakdown, demyelination and clearance of debris that occurs distal to the site of nerve injury. Wallerian degeneration is much faster, more robust, and more complete in the PNS compared to the CNS [68], and this may be a factor that contributes to better axon regeneration in the PNS. Axonal membrane damage from traumatic injury triggers Ca^2+^-mediated proteolytic activity that results in axonal breakdown [69], which orchestrates a phenotypical change in mSCs: reversion to a more immature nmSC phenotype, which in turn initiates proliferation [70]. Myelin breakdown and clearance takes place in two distinct phases. In the first phase, myelin breaks down into ovoid-shaped fragments within the cytoplasm of SCs, which clear the myelin fragments in an autophagocytic process that is the dominant function during the first 5–7 days post injury [71]. In the second phase, due to break down in the perineurial architecture distal to the nerve injury, the once impenetrable blood-nerve-barrier becomes porous and allows haematogenous macrophages to enter the injury site [70]. Where macrophages are attracted by the inflammatory chemokines (prostaglandins & leukotrienes) that are by-products of myelin degradation, and chemokines (TNF-α, LIF, MCP and others) secreted by the more abundant nmSCs [71]. SCs degrade and clear 40%–50% of the myelin debris within the first phase (<7 days post injury) through autophagy. In the later phase, the remaining myelin debris is cleared primarily by macrophage-mediated phagocytosis. Thus, clearance of axonal debris differs drastically between the olfactory nerve, where glia (OECs) mediate the majority of debris phagocytosis, and the general PNS, where more debris is cleared by macrophages (Figure 3). Interestingly, nmSCs also aid in clearing the remaining myelin debris, but this time via phagocytosis [72]. The presence of myelin debris is an important signalling initiator during Wallerian degeneration. It contains myelin-associated protein (MAG), which is known to inhibit axon regeneration [73,74]. When MAG was knocked out in a mouse line exhibiting delayed Wallerian degeneration, axon regeneration occurred despite delayed myelin clearance [73]. Moreover, the presence of myelin debris leads to stimulation of toll-like receptors (TLRs) on the surface of nmSCs [75]. TLR stimulation leads to upregulation of TLR receptors, and to the expression cytokines i.e., monocyte chemotactic protein-1 (MCP-1) [76], which is known to recruit and activate macrophages [77]. In mouse models where various TLRs have been knocked out, macrophage influx is reduced by 15%–20% [58].

#### 5.2.3. SC’s Regulation of Inflammation/Immune Response

SCs secrete many factors to recruit macrophages and modulate Wallerian degeneration, such as LIF, TNFα, IL-1α, and IL-1β [78,79]. LIF stimulates autocrine monocyte chemoattractant protein (MCP-1) expression in SCs [80] while TNFα and interleukin (IL-1β) have been linked to increased matrix metalloproteinase (MMP-9) [81,82], which are all involved in macrophage migration and recruitment. Once macrophages have been recruited to the site of injury they will also express TNFα, IL-1α, and IL-1β [83]. This positive feedback loop increases macrophage recruitment enabling expeditious Wallerian degeneration, resulting in improved axonal regeneration and therefore functional recovery. Recruited macrophages also secrete mitogens that promote nmSC proliferation [64]. Regulation of the inflammatory response throughout the regeneration process is crucial; in particular, the mitigation of the inflammatory response is important to preserve healthy tissue. During the initial 3 days after peripheral nerve injury the expression of IL-10; an anti-inflammatory cytokine, is decreased, then expression increases after 7 days post-injury and can remain above baseline levels for up to 28 days in the distal nerve stump [69,79]. Thus, this secretory pattern appears to initially promote inflammation and then diminish inflammation before it becomes deleterious. In healthy nerves, both SCs and fibroblasts express IL-10 but during Wallerian degeneration the major source of IL-10 is by haematogenous macrophages [69], suggesting that macrophages modulate their own inflammatory phenotypes and responses after peripheral nerve injury.

#### 5.2.4. SC Growth-Support Signalling

Similarly to the primary olfactory nervous system, SCs of the general PNS alter expression of neurotrophic factors following injury or stress [84] (Figure 3). SCs express low levels of NGF but levels increase sharply following injury, the same can be seen with BDNF although the increase is more gradual [84,85]. NT-3, ciliary neurotrophic factor (CNTF) and GDNF are also all found to be expressed by SCs although expression varies with nerve injury [86]. The secretions of these factors have the potential to directly and indirectly support neuron growth through autocrine action creating a more supportive phenotype and paracrine action directly affecting neuron growth. The main neurotrophin family NGF, BDNF, and NT-3 act on TrkA, TrkB and TrkC receptors respectively with a degree of affinity crossover between receptors and low affinity with p75NTR. SCs also express TrkB and TrkC, which are thought to regulate SC migration through BDNF and NT-3 [87,88]. NRG1 binding to ErbB receptors also acts as a pro-survival stimulus in neurons [26]. Finally, neurotrophins secreted from SCs have also been shown to be integral to maintaining PNS architecture and maintaining the adult phenotype of neurons [89,90].

## 6. Use for Neural Regenerative Therapies

### 6.1. OEC Transplantation

Transplantation of OECs is emerging as a promising approach for treating injuries to both the PNS and the CNS. OEC transplantation has led to successful outcomes in both animals and humans with spinal cord injury. Animal studies of spinal cord injuries showed that OECs can survive and migrate long distances into the injury site [55,91], reduce scar tissue [92] and cavity formation [93], restore breathing and climbing function [94,95] and improve hindlimb mobility [96,97]. The migratory properties of OECs into scar tissue have been attributed to their rapidly moving lamellipodial protrusions [24,98] and their ability to interact with astrocytes [99]. A recent review and meta-analysis of 62 transplantation studies in animals showed an average improved locomotor function of 20% [100]. Thus, although functional outcomes of OEC transplantation are variable, the method shows strong potential. A human phase I clinical trial showed that autologous transplantation of OECs into human spinal cords is safe [101]. Since then, many pre-clinical and clinical trials with OECs have been performed with a high variability in outcome from no improvement to remarkable functional recovery [101,102]. The most dramatic outcome was seen in a recent study of a patient with severe thoracic spinal cord transection who regained partial sensory and motor functions of the lower extremities along with improved trunk stability [103].

The reasons for the discrepancies in results are that the source (olfactory nerve versus olfactory bulb), purity and delivery method of the cells vary considerably between studies [3,104]. Culturing OECs to high purity can be difficult particularly when autologous transplantation is used and the times between biopsy collection and transplantation needs to be minimised in order to maintain the quality of the cells. OECs from the human mucosa have been reported to provide high purity cultures [101] while OECs from the human olfactory bulb tend to grow faster but may result in cultures of lower purity [95]. Thus the amount of cells and the purity of cells can vary depending on the origin of the OECs. Typically cell preparations are microinjected into the injury site of the spinal cord but the total number of cells and location of injections vary with each study. A recent study has shown the best restoration of function in humans to date [103], using a nerve bridge obtained from a sensory nerve that was implanted into the injury site together with the microinjected OECs harvested from the olfactory bulb. Thus while the use of OECs shows promise, clearly it is crucial that optimal preparation and transplantation protocols are established.

OEC transplantation has led to increased re-myelination of demyelinated tracts in the brain and/or spinal cord in animals, thus having implications for treatment of multiple sclerosis [105]. It still remains unclear however; whether OECs, which do not myelinate axons in their natural environment can myelinate axons in the target tissue, or whether they stimulate other myelinating glia (oligodendrocytes in the CNS; SCs in the PNS). OECs have also been used for peripheral nerve repair in animals. OECs survive and integrate well at the site of injury in the sciatic nerve, resulting in remyelination, increased conduction velocity, and a greater functional recovery [106]. One study, however, demonstrated more pronounced regeneration with transplantation of SCs than OECs to the injured sciatic nerve [107]. OEC transplantation to the rhizotomised dorsal root, where sensory nerves enter the spinal cord, has been less successful; although OECs integrated well and appeared to form a bridge across the lesion [108], several studies however, showed no anatomical or functional improvements [109]. Whilst many studies have focussed on the vagus nerve as a model for OEC transplantation, OECs have showed mixed outcomes in animal models of optic nerve injury, with one study showing no effect [110] and another showing that when combined with GDNF, OEC transplantation promoted axonal extension and improved nerve conduction [111]. Moreover, OECs strongly promoted axonal extension from retinal ganglion neurons in vitro [112], and ensheathed retinal ganglion axons after transplantation into the rat retina [113], reducing the detrimental Müller glia response in a rat model of retinitis pigmentosa [114].

In summary, OEC transplantation is a very promising and exciting approach for a range of neuronal injuries but results remain highly variable and their methods need improvement. Thus, optimisation and standardisation of OEC transplantation is highly warranted.

### 6.2. SC Transplantation

In significant peripheral nerve injuries, where substantial loss of nervous tissue (>2 cm) has been sustained, the surgical standard repair is an autologous nerve graft [115]. However, there is a limited amount of nerve tissue that can be harvested for such nerve reconstruction. Therefore, an alternative to nerve autografts is the use of conduits. It has been demonstrated that artificially designed conduits seeded with in vitro cultured SCs result in axon regeneration with a significantly higher fibre diameter than the surgical standard autografts [116]. Natural conduits (veins) that were filled with SCs in two large nerve gap studies using rabbits demonstrated greater nerve regeneration when compared to an empty vein graft and the nerve graft control [117,118]. However, these conduits facilitated the formation of new nerve fascicles distal to the point of injury and provided axons with greater myelin density, thus producing significantly faster average conduction velocity in veins seeded with SCs compared to the nerve grafting group. Although it has been shown that exogenously added SCs to conduits are of benefit to nerve regeneration, a serious consideration to be made is that the host’s immune system appears to reject these implanted cells around week 6 post-implantation [119]. One study showed however, that this immune rejection did not result in deleterious inflammation to the nerve tissue and still produced a more desired outcome when compared to autologous harvested SCs [119].

Many studies have shown that transplanted SCs can promote spinal cord repair after injury, by mediating axonal regeneration, scar tissue mitigation and remyelination [120]. Transplantation of SCs into rat spinal cord contusions were more successful when compared to other glia, in terms of promoting axonal regeneration and re-myelination, less cavitation, and ultimately greater hindlimb locomotion [120]. However, two relatively recent studies concluded that SC transplantation alone produced only modest functional recovery and axonal re-myelination when compared to co-cultured transplants and those with additional growth factor support [121,122]. Interestingly, one study concluded that SCs migration and re-myelinating activity was restricted to areas where astrocytes were absent [123]; suggesting that the behaviour of transplanted SCs are dependent on the microenvironment at the transplantation site. Furthermore, a number of studies have shown that SCs transplanted in combination with other cells (Section 6.3), neuroprotective/neurotropic factors and inhibitory molecules produce more favourable outcomes.

As mentioned previously, SCs have the potential to re-myelinate damaged axons when transplanted into the CNS. In a clinical trial of multiple sclerosis (MS) patients, transplanted SCs were considered to be safe and promoted remyelination [124], however, a phase 1 clinical trial in 2001–2002 using SCs transplantation in MS patients was discontinued as there was no evidence that the implanted SCs remained viable. SCPs were used more recently in an animal CNS transplantation study; SCPs survived, migrated and interacted with CNS glia well and remyelinated injured axons extensively, when compared to SC only transplants [125].

### 6.3. Co-Transplantation of OECs and SCs

Transplantation of a combination of OECs and SCs has recently emerged as an alternative approach to transplantation of each cell type independently. It is possible OECs promote SC-mediated myelination, or that the two cell types stimulate each other’s regenerative properties in other manners. In vitro studies have shown that OECs promote SC migration [126], and secrete a protein termed SPARC (secreted protein acid rich in cysteine), which potentiates SC-mediated DRG neuron extension [127]. In an animal model of sciatic nerve injury, OECs and SCs transplanted together resulted in superior outcomes than the two cell types applied alone [128]. OECs have been co-transplanted with a SC “bridge” and chondroitinase (ChABC) into the injured spinal cord, resulting in significantly increased numbers of both myelinated axons in the SC bridge and serotonergic fibres that grow through the bridge improving forelimb and hindlimb movements as well as open-field locomotion, compared with grafts only or the untreated control [121]. One study showed that a combination of a SC-infused channels in a transected spinal cord using a rodent model in conjunction with OECs resulted in superior regeneration of axons when compared to a channel seeded with SCs alone [129]. One study in 28 patients with spinal cord injury showed that combined transplants of OECs and SCs were well tolerated [130], but it remains to be investigated whether a combination of OECs and SCs yield better outcomes than transplantation of each cell type alone.

## 7. Conclusions

Numerous studies show that SCs and OECs are strong candidates for transplantation therapies targeting neural injuries and neurodegenerative disorders. Future research must be focussed on optimising isolation, purification and delivery of the cells. Particular focus should be placed on choosing the most optimal matrices to create three-dimensional cell bridges, and evaluating the impact of co-transplantation of different cell types, such as OECs and SCs combined. A thorough characterisation of how the cells are modulated by growth factors and signalling molecules are highly warranted so that cell behaviour following transplantation can be accurately manipulated.
